# A Polyelectrolyte Complexation Strategy Enabling Tough and Absorbent Chitosan-Based Xerogels via Simple Atmospheric Drying

**DOI:** 10.3390/foods15071132

**Published:** 2026-03-25

**Authors:** Jiangyang Su, Sijing Liang, Ouyang Zheng, Zongyuan Han, Naiyong Xiao, Yantao Yin, Shucheng Liu, Qinxiu Sun

**Affiliations:** 1 Guangdong Provincial Key Laboratory of Aquatic Product Processing and Safety, Guangdong Province Engineering Laboratory for Marine Biological Products, Guangdong Provincial Engineering Technology Research Center of Seafood, Key Laboratory of Advanced Processing of Aquatic Product of Guangdong Higher Education Institution, College of Food Science and Technology, Guangdong Ocean University, Zhanjiang 524088, China; 19197143181@163.com (J.S.); liangsijing0912@163.com (S.L.); zhengouyang07@163.com (O.Z.); longnv0206@163.com (Z.H.); nyxiao@gdou.edu.cn (N.X.); yantaoyin@gdou.edu.cn (Y.Y.); liusc@gdou.edu.cn (S.L.); 2 Collaborative Innovation Center of Seafood Deep Processing, Dalian Polytechnic University, Dalian 116034, China

**Keywords:** chitosan, xerogel, polyelectrolyte complex, water absorption rate, mechanical strength

## Abstract

The structure collapse and performance degradation caused by traditional air-drying technology often hinder the practical application of bio-based xerogels as absorbent pads. In this study, chitosan (CS) and different types of polyanions (carboxymethyl cellulose (CMC), sodium alginate (SA), hyaluronic acid (HA), pectin (PT) and xanthan gum (XG)) in different proportions were used to prepare an xerogel resistant to atmospheric pressure air drying collapse, and its potential as an absorption pad was systematically evaluated. The results showed that among all the treatments, CS/CMC xerogel at an optimal mass ratio of 1:3 demonstrated superior comprehensive properties. It exhibited minimal shrinkage (*p* < 0.05) and high porosity, coupled with an exceptional water absorption capacity (140% higher than CS/PT) and hardness (96% higher than CS/SA and CS/HA). FTIR and XRD revealed that strong electrostatic interactions and potential amide bond formation between CS and CMC resulted in a dense yet homogeneous network with low crystallinity. SEM imaging further corroborated a uniform thin-walled porous structure. This stable network contributed to high toughness, of CS/CMC significantly surpassing the brittle CS/XG and CS/PT xerogels (*p* < 0.05). CS/CMC xerogel is an ideal absorbent material with high absorption, stability, and controllable structure.

## 1. Introduction

During storage and transportation at 0–4 °C, chilled fresh meat experiences exudation of drip loss due to the degradation of myofibrillar structures and damage to cell membranes [1]. This not only results in weight loss but also creates an ideal environment for microbial proliferation, directly leading to food safety risks and waste [1]. High-performance absorbent pads are critical for maintaining meat quality. However, commercially available polyethylene-based pad materials, which are difficult to biodegrade, suffer from inherent drawbacks such as limited absorption efficiency and potential leakage of absorbed fluids [2]. There is an urgent need to develop biodegradable and efficient alternatives.

Xerogels feature a highly porous three-dimensional network and large specific surface area, enabling efficient moisture immobilization through capillary action [3,4]. They also serve as a platform for loading functional molecules (e.g., antimicrobials), offering potential active preservation functionality. Among them, natural polysaccharide-derived xerogels are particularly promising for food packaging due to their excellent biocompatibility and safety. However, the macroscopic properties of xerogels depend heavily on the drying technique. While supercritical drying and freeze-drying effectively preserve the gel network structure, they entail high costs. In contrast, atmospheric drying is cost-effective but struggles with network collapse caused by strong capillary forces during process [5].

Constructing a robust gel network is crucial for resisting capillary stresses during drying. As a green and efficient physical cross-linking strategy, polyelectrolyte complexation enables the spontaneous formation of a stable three-dimensional network through electrostatic attraction between oppositely charged polyelectrolytes [6]. Chitosan (CS), a natural polycation, possesses amino groups that protonate and carry a positive charge under acidic conditions, allowing it to complex with polyanions, thereby forming a stable network structure [7]. The type and content of the polyanion are critical factors influencing the strength and toughness of the complexed gel network [8]. Although chitosan–polyanion systems have been extensively studied, existing research predominantly focuses on specific applications, such as drug delivery carriers or heavy metal adsorption. How to achieve a balance of high mechanical strength, rapid liquid absorption, low hysteresis, and structural stability through the precise selection remains unclear.

In this study, a series of xerogels were prepared using CS as the polycation together with carboxymethyl cellulose (CMC), sodium alginate (SA), xanthan gum (XG), pectin (PT), and hyaluronic acid (HA) at varying mass ratios. The mass ratio of CS to each polyanion was first optimized by evaluating key parameters such as shrinkage rate, water absorption capacity, and hardness. Based on the optimized ratios, xerogel samples with different compositions were produced. The microstructure, molecular interactions, water absorption and retention properties, as well as mechanical characteristics of the various groups, were subsequently investigated, providing a foundation for developing high-performance biodegradable xerogel materials.

## 2. Materials and Methods

### 2.1. Chemical Reagent

All reagents and chemicals were of analytical grade. Ethanol (purity ≥ 99.7%), acetic acid (purity ≥ 99.0%,), calcium chloride (purity ≥ 99.0%) and potassium bromide (SP, purity ≥ 99.9%) were bought from Sinopharm Chemical Reagent Co., Ltd. (Shanghai, China). CS (purity > 95%, molecular weight: <1000 Da, 95% degree of deacetylation (DD)) was purchased from Guangdong Kangda Biotechnology Co., Ltd. (Guangzhou, China); PT (purity ≥ 99.0%, Mw = 280 kDa, galacturonic acid ≥ 66.2%, degree of esterification ≥ 55.0%) was purchased from Xinjiang Fufeng Biotechnology Co., Ltd. (Urumqi, China); XG (purity ≥ 99.0%, molecular weight: 241 kDa, propionylation degree 10.13% (Molar ratio), acetylation degree 0.39% (Molar ratio)) was purchased from Xinjiang Meihua Amino Acid Co., Ltd. (Urumqi, China); SA (purity ≥ 99.0%, 1% viscosity: 7000–10,000 mPa·s, molecular weight: 300–500 kDa, M/G = 1:1) was purchased from Qingdao Mingyue Seaweed Group Co., Ltd. (Qingdao, China); HA (purity ≥ 99.0%, molecular weight: 200–400 kDa) was purchased from Xinjiang Fufeng Biotechnology Co., Ltd. (Urumqi, China); CMC (purity, ≥99.0%, molecular weight: 250,000 g/mol, degree of substitution: 0.9, viscosity: 2500–3100 mPa·s) was purchased from Shanghai Shenguang Food Chemicals Co., Ltd. (Shanghai, China).

### 2.2. Preparation of Chitosan/Polyanion Xerogel

The CS/polyanion xerogels were prepared following methods adapted from Druel et al. [9] and Tang et al. [10]. A 2% (*w*/*v*) CS solution was prepared by dissolving CS in a 2% (*w*/*v*) acetic acid aqueous solution under ultrasonication. Each polyanion (SA, HA, CMC, PT, and XG) was then mixed into the CS solution to achieve CS/polyanion mass ratios (*w*/*w*) of 1:2, 1:2.5, 1:3, 1:3.5, and 1:4. The mixture was induced by stirring the mixture at 400 rpm for 30 min in a 90 °C water bath, ensuring uniform cross-linking. The resulting dispersion was then poured into a plastic mold with dimensions of 10 cm × 7 cm × 1.2 cm, and then frozen at −80 °C for 6 h until completely frozen. The resulting hydrogel was removed from the mold and subjected to solvent exchange by immersion in a five-fold volume of anhydrous ethanol. The exchange was performed three times, each lasting 3 h, yielding an alcogel. Finally, the alcogel was dried at 26 °C, 40% relative humidity for 48 h to obtain the xerogel.

### 2.3. Shrinkage Ratio

The shrinkage ratio of the xerogels was determined according to the method described by Machado et al. [11]. A flat glass plate was used as a reference plane. The length, width, and height of the xerogel samples were measured at corresponding positions using a high-precision vernier caliper, and the volume was calculated. The shrinkage ratio was then determined using the following formula:

(1) $$\mathrm{Shrinkage}\;\mathrm{ratio}=\left(\frac{V_{i}-V_{f}}{V_{i}}\right)\times 100\%$$

where *V_i_* (cm^3^) is the initial volume of the gel before drying (i.e., the volume of the mold cavity or the wet gel after demolding). *V_f_* (cm^3^) is the final volume of the gel after drying.

### 2.4. Porosity

The porosity of the xerogels was determined following the method described by Shiri et al. [12]. A square xerogel sample measuring 20 mm × 20 mm × 5 mm was cut, weighed, and its mass was recorded. Subsequently, the sample was immersed in 50 mL of anhydrous ethanol within a centrifuge tube, and left to soak at 26 °C for 24 h. After soaking, the weight of the sample was measured again. The porosity was calculated using the following formula:

(2) $$Pr=\left(\frac{M_{1}-M_{0}}{\rho V}\right)\times 100\%$$

where *Pr* is the porosity of the xerogel. *M*_1_ (g) is the initial mass of the xerogel sample. *M*_0_ (g) is the mass of the xerogel sample after immersion. *ρ* (g/cm^3^) is the density of the immersion liquid (absolute ethanol) at the measurement temperature. *V* (cm^3^) is the geometric volume of the xerogel sample.

### 2.5. Compressive Properties

The compressive properties of the xerogels were evaluated according to the method described by Rizal et al. [13]. TA. XT plusC texture analyzer (Stable Micro Systems Inc., Godalming, UK) with a 5 kg load cell was used for the compression tests. A 20 mm × 20 mm × 5 mm sample was cut from xerogel. A texture profile analysis (TPA) test was performed using a P/36R probe (diameter 36 mm). The pre-test speed, test speed, and post-test speed were set to 3.0 mm/s, 1.0 mm/s, and 3.0 mm/s, respectively. The compression ratio was set to 50%, with a trigger force of 5 g. At least six samples of each xerogel formulation were tested.

### 2.6. Tensile Properties

The tensile properties of the xerogels were evaluated with reference to Cao et al. [14]. Samples were cut to the standard dimensions of 25.4 mm × 5 mm × 0.5 mm. The samples were cut into a rectangular spline with a length of 30 mm and a width of 5 mm, and the thickness was accurately measured with a vernier caliper. TA.XT plusC texture analyzer (Stable Micro Systems Inc., Godalming, UK) was used to perform the tensile tests at a speed of 2 mm/min until 80% strain was reached. The slope of the line connecting the initial point and the fracture point on the stress–strain curve is taken as the modulus. Calculate the EAB based on the results. The principal formula is given as follows:

(3) $$EAB=\frac{\Delta L}{L}\times 100\%$$

where *L* is the original length of the material and Δ*L* is the displacement length of the material after stretching.

### 2.7. Water Absorption Capacity

The water absorption capacity (WAC) of the xerogels was determined following the method described by Li et al. [15]. A 20 mm × 20 mm × 5 mm sample was cut from xerogel, weighed, and then immersed in a sealed container containing 50 mL of distilled water. After soaking at 4 °C for 8 h, the sample weight was measured. The WAC was calculated as follows:

(4) $$WAC=\left(\frac{W_{s}-W_{d}}{W_{d}}\right)\times 100\%$$

where *W_s_* (g) is the weight of the xerogel sample after 8 h immersion. *W_d_* (g) is the initial weight of the xerogel sample.

### 2.8. Water Retention Capacity

The water retention capacity of the xerogel was determined according to the method described by Gao et al. [16]. The xerogel samples were cut into squares 20 mm × 20 mm × 5 mm. For the test, the xerogel samples were first immersed in deionized water at 26 °C for 8 h, after which their weight was recorded. Subsequently, the samples were placed in a desiccator with a saturated calcium chloride solution (providing a relative humidity of approximately 30%) acting as a desiccant. The samples were weighed every 3 d, and the measurement continued for a total of 15 d. The water retention ratio, *P* (%), was calculated using the following formula:

(5) $$P=\left(\frac{M_{n}}{M_{0}}\right)\times 100\%$$

where *M_n_* (g) is the mass of the hydrogel dressing sample measured on the *n*th day in the desiccator. *M*_0_ (g) is the initial mass of the hydrogel dressing sample after saturation.

### 2.9. Swelling Ratio

The swelling ratio of the xerogels in water was investigated using a gravimetric method according to the procedure described by Huang et al. [17]. Square xerogel samples (20 mm × 20 mm × 5 mm) were dried repeatedly in oven at 60 °C until a constant weight was achieved, and their dry weight (*W_d_*) was recorded. The xerogels were then completely immersed in deionized water. The swelling ratio was measured at predetermined intervals. After gently blotting away excess surface water from the samples, the swollen weight (*M_t_*) was recorded. The swelling ratio (*Sr*) was then calculated as follows:

(6) $$Sr=\left(\frac{M_{t}}{M_{0}}\right)\times 100\%$$

where *M*_0_ (g) is the initial dry mass of the xerogel sample. *M_t_* (g) is the wet mass of the gel sample at time *t*.

### 2.10. Water Vapor Adsorption Capacity

The water vapor adsorption capacity (WVSC) was determined following the method described by Zhou et al. [2]. A square xerogel sample measuring 20 mm × 20 mm × 5 mm was prepared, and its initial dry weight was recorded. The sample was then placed in a sealed container maintained at 26 °C with water to create a 100% relative humidity environment for equilibration over 48 h. After the sample reached constant weight, the final weight (*W_t_*) was measured using an analytical balance. The water vapor adsorption capacity (WVAC) was calculated as follows:

(7) $$WVSC=\left(\frac{W_{m}-W_{t}}{W_{n}}\right)\times 100\%$$

where *W_m_* (g) is the weight of the xerogel sample after 24 h equilibration under 100% relative humidity at 26 °C. *W_n_* (g) is the initial dry weight of the xerogel sample.

### 2.11. Fourier Transform Infrared

The functional groups of the samples were characterized by TENSOR27 Fourier transform infrared (FTIR) spectroscopy (Bruker Inc., Karlsruhe, Germany). FTIR spectroscopy was conducted following the method described by Lan et al. [18]. Prior to the FTIR analysis, the xerogel samples were ground into a fine powder using an agate mortar and dried in an oven at 105 °C for 2 h. For measurement, the dried sample was mixed with potassium bromide (KBr) at a mass ratio of 1:200, and finely ground again in an agate mortar. The homogeneous mixture was then compressed into a transparent pellet. The FTIR spectra were recorded in the wavelength range of 4000 to 400 cm^−1^ with a resolution of 4 cm^−1^ and 32 scans.

### 2.12. X-Ray Diffraction

X-ray diffraction (XRD) analysis was performed according to the method described by Li et al. [19]. Approximately 50 mg of finely ground xerogel powder was used for the analysis. The measurement was conducted using an X’Pert3 Powder X-ray diffractometer (PANalytical Inc., Almelo, The Netherlands) with Cu *K*_α_ radiation. The scanning range was from 10° to 80° (2*θ*) with a scanning rate of 4°/min.

### 2.13. Scanning Electron Microscopy

Scanning electron microscopy (SEM) analysis was conducted according to the method described by de Oliveira et al. [20]. The xerogel samples were frozen in liquid nitrogen and fractured. The fractured samples were mounted on conductive adhesive and sputter-coated with a gold–palladium mixture for 3 min under vacuum before examination. The surface and cross-sectional morphology of the xerogel samples were observed using a XL30 scanning electron microscope (Philips Inc., Eindhoven, The Netherlands) under an accelerating voltage of 5 kV and a working distance of 19 mm.

### 2.14. Statistical Analysis

All experiments were performed with at least three independent replicates (*n* ≥ 3) per condition. Data are presented as mean ± standard deviation (mean ± SD). Statistical analysis was performed using SPSS software (v19.0). Data meeting the assumptions of normality and homogeneity of variances were subjected to one-way analysis of variance (ANOVA) to test for significant differences among treatment groups. Where ANOVA indicated a significant difference, Tukey’s honestly significant difference post hoc test was applied for pairwise comparisons. The significance level was set at *p* < 0.05. All figures were generated using Origin software (v2022).

## 3. Results and Discussion

### 3.1. Shrinkage Ratio and Water Absorption Capacity

**Figure 1:**
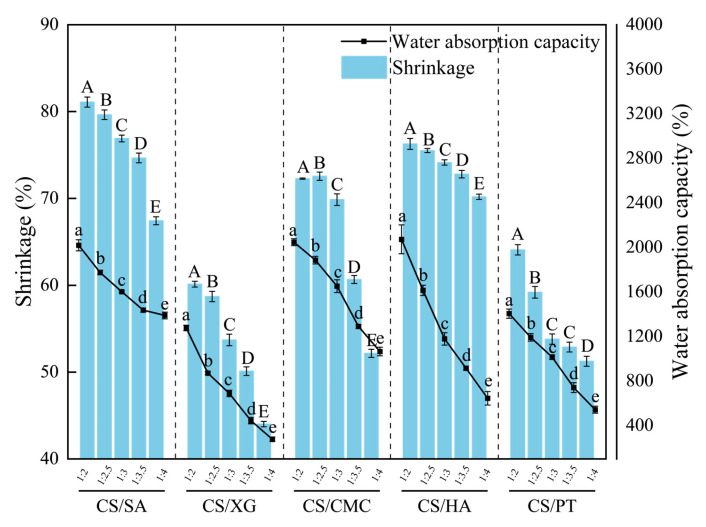
Variations in shrinkage ratio and water absorption capacity of xerogels with different polyanion ratios. Different capital letters denote statistically significant differences in the shrinkage ratio among different groups (*p* < 0.05), whereas different lowercase letters indicate statistically significant differences in the water absorption among different groups (*p* < 0.05). (CS/SA: CS-SA xerogel; CS/XG: CS-XG xerogel; CS/CMC: CS-CMC xerogel; CS/HA: CS-HA xerogel; CS/PT: CS-PT xerogel).

The shrinkage of the xerogels reflects the stability of their internal network; less shrinkage usually indicates stronger structural integrity and better resistance to collapse during drying [21]. Its porosity is influenced by factors such as the stiffness of molecular chains, chain architecture, and interactions between polymer chains and solvent molecules [22]. As shown in Figure 1, the shrinkage rate of the xerogels in all groups decreased as the proportion of polyanion increased, indicating that the introduction of polyanions effectively enhanced the material’s resistance to drying-induced stresses. This phenomenon can be elucidated through the lens of intermolecular forces. The chitosan–polyanion composite system is governed by three primary types of interactions: intra- and intermolecular hydrogen bonds within CS, where the hydroxyl group at the C3 position of the CS molecular chain can form intramolecular hydrogen bonds with the glycosidic oxygen of an adjacent residue or intermolecular hydrogen bonds with sugar ring oxygens on neighboring chains [23]; electrostatic repulsion and hydrogen bonding between polyanion segments, where negatively charged groups generate intermolecular electrostatic repulsion, partially counteracted by hydrogen bonds between polar groups such as hydroxyl and carboxyl [24]; and finally the electrostatic attraction and hydrogen bonding between the protonated amino groups of CS and the anionic groups of the polyanions. The relative strength of these interactions typically follows the order chitosan–polyanion > chitosan–chitosan > polyanion–polyanion.

This may be due to the fact that, at low polyanion ratios, hydrogen bonding between CS chains predominated, leading to the formation of local aggregation zones and a heterogeneous microstructure [4]. During drying, these regions were more susceptible to chain slippage and densification, resulting in greater volumetric shrinkage [25,26]. Within a certain proportion threshold range, as the proportion of polyanion increases, the electrostatic cross-linking density between the polyanion and chitosan increases, gradually replacing the tendency of chitosan to self-aggregate [27]. This promoted a more uniform molecular chain distribution and enhanced the stability of the cross-linked network, effectively suppressing drying shrinkage [28]. For treatments with the same polyanion addition ratio, CS/SA exhibited the highest shrinkage, followed by CS/HA, CS/CMC, and CS/PT, with CS/XG showing the lowest shrinkage (*p* < 0.05). This may be explained by structural differences between polyanions. SA contains only one carboxyl group per mannuronic (M) or guluronic (G) unit: the G unit, with its *α*-configured hydroxyl at C1 [29]. This conformation restricts the spatial exposure of carboxyl groups, reduces the effective negative charge density, and leads to a sparser distribution of anionic sites, thereby weakening electrostatic interactions and stress-dissipation capacity [30]. In contrast, CS/HA, CS/CMC, and CS/PT possess higher negative charge densities and greater molecular chain rigidity, contributing to their lower shrinkage. The pyruvate and acetate groups on the side chain of XG molecule restricted the free rotation of the main chain through the steric hindrance effect; therefore, the chain rigidity of XG was the strongest [31]. Although this rigidity may limit its cross-linking efficiency, the inherent stiffness of XG effectively counteracts capillary forces during drying, resulting in the lowest observed shrinkage rate.

The water absorption capacity of the xerogels is a key functional indicator for their use as meat preservation pads, as it directly affects their ability to absorb moisture and regulate humidity in packaging [32]. As shown in Figure 1, the water absorption capacity of the xerogels in all groups gradually decreased as the proportion of polyanion increased (*p* < 0.05). This decrease was primarily attributed to two kinds of structural changes. On the one hand, polyanion segments infiltrated and filled the interstices within the chitosan-based three-dimensional network, increasing the apparent density of the material and consequently reducing the total pore volume [33]. Therefore, the water absorption capacity of the sample was reduced. On the other hand, while the introduction of polyanions increases hydrophilic groups and enhances overall hydrophilicity, their high content can induce rapid surface swelling upon water absorption. This swelling not only significantly increases the system’s viscosity [34], but also obstructs the connectivity between internal pores and external water. Consequently, the dominant mechanism of water ingress shifts from rapid capillary-driven penetration to slower diffusion-controlled transport, substantially reducing the overall water absorption capacity. At the same mass ratio, CS/SA exhibited the highest water absorption capacity among other samples, which can be attributed to its relatively loose cross-linked network and pronounced swelling ability, enabling significant expansion of water-holding space during absorption. CS/CMC and CS/HA demonstrated more balanced water absorption performance. Their moderate cross-linking density allowed them to maintain better pore inter-connectivity during swelling, achieving a compromise between water absorption capacity and structural stability. In contrast, CS/PT and CS/XG showed lower water absorption capacity, particularly at higher polyanion ratios, where their greater molecular chain rigidity and steric hindrance effects limited network expansion.

### 3.2. Hardness

**Figure 2:**
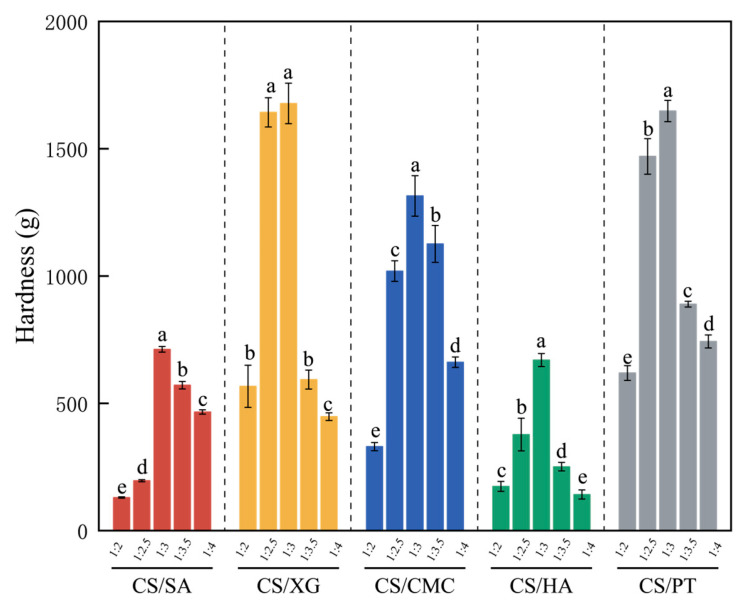
Variation in xerogel hardness with different polyanion ratios. Different lowercase letters indicate statistically significant differences in the water absorption among different groups (*p* < 0.05).

The hardness of the xerogel directly relates to the material’s ability to resist compression and prevent deformation or rupture during storage and transportation—both critical for maintaining packaging integrity [32]. Its hardness is influenced by multiple factors, including skeleton interactions, the number of cross-linking sites, porosity, and others [19]. As shown in Figure 2, the hardness of all five CS/polyanion xerogels initially increased and then decreased as the amount of polyanion added increased, reaching a maximum at a CS-to-polyanion ratio of 1:3 (*p* < 0.05). This change is related to the formation mechanism of CS–polyanion cross-linked network. At low polyanion addition levels, the insufficient number of polyanions resulted in a low density of electrostatic cross-linking points. The network is primarily governed by weaker hydrogen bonding, making it difficult to form a stable network structure, which led to relatively low hardness. When the ratio increases to 1:3, a high-density, uniform cross-linked network forms between CS and the polyanion through electrostatic interactions and hydrogen bonding, effectively distributing external stress and resulting in maximum hardness. However, when the polyanion is in excess, the free polyanions present in the system can competitively bind with the amino groups on CS molecular chains, thereby interfering with effective cross-linking reactions. Simultaneously, an excessively high cross-linking rate tends to result in an inhomogeneous cross-linked network, creating structural defects characterized by localized over-cross-linking and insufficient cross-linking in other regions, which induces stress concentration and ultimately reduces the overall hardness of the material [35]. This phenomenon aligns with the general principle of cross-linking agent behavior, whereby the performance of the material initially improves and then declines as the cross-linking agent content increases, indicating the existence of an optimal threshold.

At a mass ratio of 1:3, CS/XG and CS/PT exhibited the highest hardness, followed by CS/CMC, whereas CS/SA and CS/HA showed the lowest values. This difference in hardness is primarily due to the relatively rigid molecular chains of CS/XG and CS/PT, which form hydrogels with a compact structure and low porosity, leading to their higher hardness [19]. In contrast, CS/SA and CS/HA exhibit relatively lower hardness, which can be attributed to their higher porosity and looser structure. Although CS/CMC also possesses considerable porosity, the inherent toughness of the cellulose molecular chains helps reinforce the network structure [36,37], thereby yielding an intermediate level of hardness between the two aforementioned groups. The variation trend of hardness is inconsistent with that of shrinkage, which may be attributed to the fact that when shrinkage is high, porosity is relatively low, and the influence on the degree of molecular chain cross-linking predominates. As shrinkage increases, the porosity of the gel rises, and the effect of pore structure on hardness becomes more pronounced, leading to a reduction in hardness [38,39].

### 3.3. Discussion

The shrinkage, water absorption capacity, and hardness of CS-based xerogels are co-regulated by the type of polyanion and the CS-to-polyanion mass ratio. Within the mass ratio range of 1:3 to 1:4, the CS/XG system exhibited the lowest shrinkage, whereas CS/CMC maintained relatively low shrinkage along with a more balanced network cross-linking density. In the range of 1:2 to 1:3, CS/SA achieved the highest water absorption due to its pronounced swelling capacity, while CS/CMC demonstrated favorable comprehensive application potential by retaining good water absorption together with structural stability. At the optimal ratio of 1:3, CS/XG and CS/PT reached the highest hardness, while CS/CMC preserved a favorable balance between hardness, water absorption capacity, and mechanical properties. Based on the overall performance assessment, a CS/CMC mass ratio of 1:3 was selected as the optimized formulation for subsequent experiments to systematically compare the influence of different polyanions on the comprehensive properties of the xerogels.

### 3.4. Surface Functional Groups

**Figure 3:**
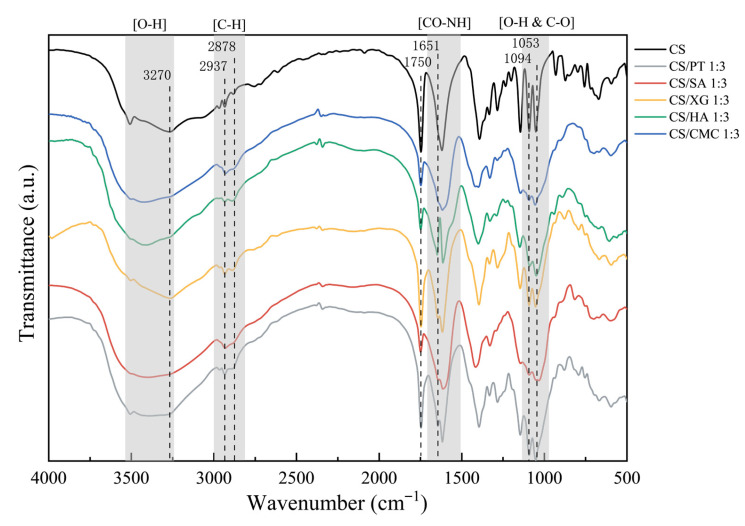
Infrared spectra of CS and five types of xerogels.

FTIR provided crucial molecular-level evidence for elucidating the formation mechanism and stability variations in the xerogel network structures. As shown in Figure 3, spectroscopic analysis revealed that the CS exhibits a broad, strong absorption peak near 3270 cm^−1^, which is attributed to the overlapping stretching vibrations of O-H and N-H groups. In contrast, for all composite dry gels, the peak intensity at this location significantly decreased and shifted. This phenomenon confirms that hydrogen bonds are formed between the polyanion and CS, and the formation of new hydrogen-bonding interactions effectively weakens the original intramolecular and intermolecular hydrogen bonds within CS, thereby promoting the formation of a more uniform network structure [13]. For CS/SA, CS/CMC, and CS/HA samples, the significant weakening of the carbonyl peak near 1750 cm^−1^ and the appearance of a distinct new peak at 1651 cm^−1^ (amide) suggest that, in addition to electrostatic interactions, the carboxyl groups of these polyanions and the protonated amino groups of chitosan may have formed stable amide bonds via an amidation reaction [40,41], thus enhancing the stability of the network structure. In contrast, spectral changes in these regions were relatively weak for CS/XG and CS/PT samples, indicating that their interaction with CS relies predominantly on hydrogen bonding, with limited electrostatic or covalent cross-linking. This behavior is directly associated with the higher chain rigidity and lower accessibility of carboxyl groups in these polyanions.

In summary, all five xerogels are capable of network formation through hydrogen bonding interactions. Compared with CS/XG and CS/PT, CS/SA, CS/CMC, and CS/HA achieve stronger chemical cross-linking through amide bonds.

### 3.5. Crystal Structure

**Figure 4:**
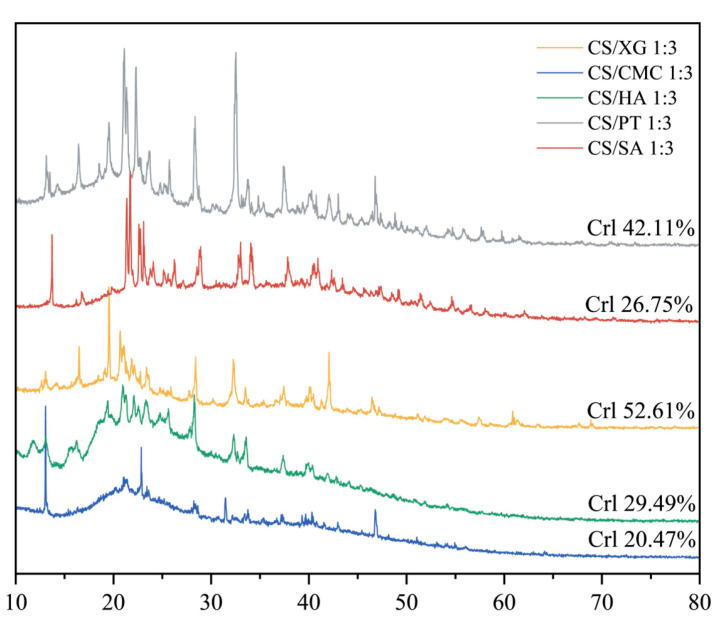
X-ray diffraction spectra of five types of xerogels.

XRD is used to analyze the crystallinity and structural order of xerogels. Broader and weaker diffraction peaks generally indicate higher amorphous content and lower crystallinity [42]. As shown in Figure 4, CS/XG and CS/PT exhibited the highest crystallinity values (52.61% and 42.11%, respectively), followed by CS/HA and CS/SA (29.49% and 26.75%), while CS/CMC (20.47%) displayed the lowest crystallinity. This variation was closely associated with the strength of interaction between CS and the different polyanions. In the CS/CMC, CS/SA, and CS/HA samples, strong electrostatic interactions and potential amide bond formation between the carboxyl groups of the polyanions and the amino groups of CS likely disrupted the inherent ordered arrangement of both components, thereby inhibiting crystal formation and leading to a significant reduction in crystallinity [43]. In contrast, for CS/XG and CS/PT samples, the higher molecular rigidity and greater steric hindrance of the polyanions limited their interaction with CS primarily to hydrogen bonding, resulting in relatively weaker cross-linking. Consequently, the disruption to the original crystalline structure was less pronounced, allowing these composites to retain higher crystallinity. A typically higher proportion of the amorphous phase generally enhances the mobility of polymer chain segments, thereby endowing the material with greater flexibility [44]. CS/CMC sample exhibited the lowest crystallinity. This largely amorphous structure contributed to greater flexibility and higher elongation at break [45]. Overall, CS/CMC sample exhibits the lowest crystallinity, which contributes to its superior adsorption capacity and flexibility, making it a promising material for applications such as food preservation pads.

### 3.6. Microstructure

**Figure 5:**
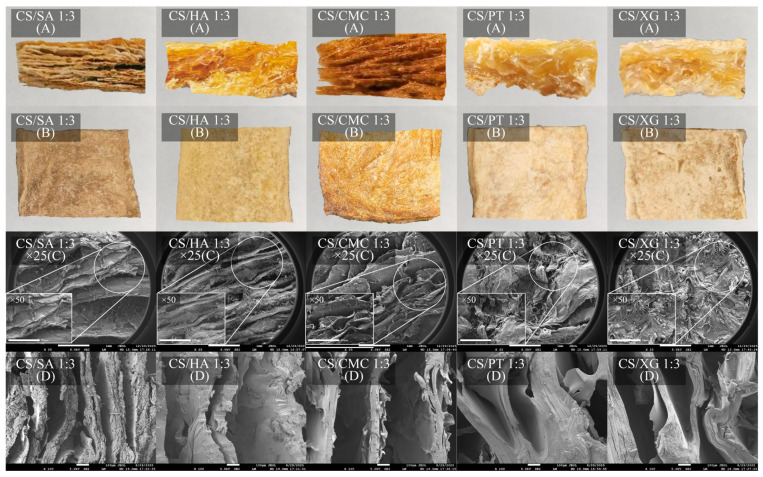
Cross-sectional images of five types of xerogels. (**A**), Sample pictures of five types of xerogels (**B**), SEM images of xerogel surface (**C**), SEM images of xerogel cross-section (**D**).

Scanning electron microscopy (SEM) is a vital analytical tool for characterizing the micromorphology and pore structure of xerogel materials. A network structure was formed via hydrogen bonds and amide bond cross-linking between CS and polyanions in an aqueous system. Subsequently, the alcogel obtained after solvent exchange undergoes rapid ethanol evaporation to yield a porous structure. The cross-sectional morphology of the samples reveals distinct differences: CS/SA, CS/HA, and CS/CMC composites are characterized by abundant, lamellar structure, whereas the CS/PT and CS/XG composites exhibit irregular and heterogeneous pore structures (Figure 5C,D). CS/SA, CS/HA, and CS/CMC samples display a coherent lamellar architecture with thin pore walls, indicative of a well-integrated 3D network (Figure 5B). CS/CMC samples exhibited a uniform pore minimum distribution and the wall thickness, which may promote water absorption and stress resistance. This morphology is closely associated with its high cross-linking density and stable network, in which strong electrostatic interactions and potential amide-bond formation contribute to a regular and robust porous skeleton. In contrast, CS/PT and CS/XG samples displayed thicker pore walls, non-uniform pore distribution, and the presence of local aggregates and denser regions, pointing to weaker cross-linking and insufficient network homogeneity [46]. Furthermore, varying amounts of hexagonal crystalline structures were observed on the surfaces of CS/SA, CS/HA, CS/XG, and CS/PT samples (Figure 5C), with crystallization being especially pronounced in CS/XG and CS/PT. This phenomenon is mainly attributed to the local enrichment and self-aggregation of polyanion molecules that did not participate in crosslinking during the freezing process. During the solvent exchange and subsequent drying stages, these unbound molecules underwent rearrangement driven by interactions such as hydrogen bonding, eventually forming ordered crystalline domains. This observation reflects incomplete cross-linking and poor component compatibility in these samples. In contrast, the surface of CS/CMC sample appeared clean, without obvious crystalline precipitates, indicating a sufficient cross-linking reaction [47]. This finding aligns with its lowest crystallinity, as observed in XRD analysis. In short, CS/CMC, characterized by uniform thin walls, large pore size, and high connectivity, demonstrated the greatest potential for liquid absorption and retention.

### 3.7. Porosity

**Figure 6:**
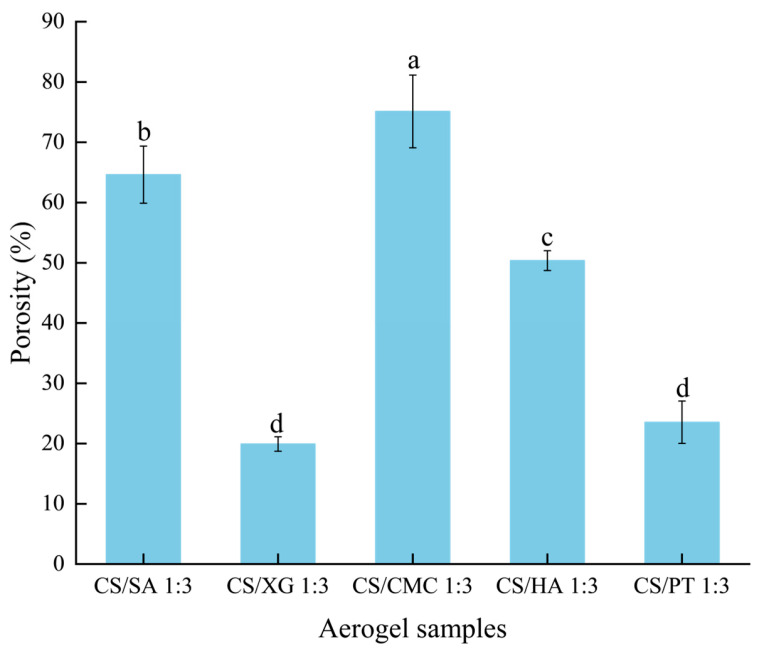
Changes in the porosity of xerogels with different types of polyanions. Different lowercase letters indicate statistically significant differences in the water absorption among different groups (*p* < 0.05).

Xerogel porosity indicates its internal packing density, with higher porosity typically correlating with lower density. This property is beneficial for producing lightweight, efficient absorbent pads [48]. As shown in Figure 6, CS/CMC sample exhibited the highest porosity, followed by CS/SA, CS/HA, and CS/PT samples, with CS/XG sample showing the lowest. This may be attributed to the fact that the carboxyl groups of CS can form strong amide bonds with the amino groups of SA, CMC and HA (Figure 3). This promotes the formation of a three-dimensional network structure characterized by high cross-linking density and stability, which effectively counteracts the destructive capillary forces generated during the drying process. As a result, the porous structure of the initial gel is better preserved. In contrast, the cross-linking in the CS/PT and CS/XG relies predominantly on hydrogen bonding and weaker electrostatic interactions (Figure 3), rendering them more susceptible to structural collapse during drying, which leads to a significant reduction in porosity. PT due to intramolecular hydrogen bonding in its side chains, and XG as a stable helical [49]. These rigid conformations exhibit limited flexibility and restricted rotation, imposing considerable steric hindrance that obstructs the close approach of other molecules or functional groups [11]. Consequently, the carboxyl groups of PT/XG and the amino groups of sufficient cross-linking and leading to significant compression of the pore space, thereby reducing porosity. Among the three groups with higher porosity (CS/SA, CS/CMC, and CS/HA), the difference in porosity can be attributed to variations in the density and accessibility of carboxyl groups on the corresponding polyanion chains. As a highly substituted cellulose derivative, CMC possesses high carboxyl group density [50]. This characteristic enables the formation of uniform and dense cross-linked network with CS, resulting in the highest porosity. In contrast, the carboxyl groups of SA are primarily located on the guluronic acid units, giving it a moderate carboxyl group density [51]. Meanwhile, the carboxyl groups of HA are present only on the glucuronic acid units, resulting in the lowest overall density. Consequently, the gel network formed by HA exhibits relatively weaker stability and pore-retention capacity [52]. In brief, CS/CMC had a three-dimensional network structure with high porosity, thereby providing an important structural foundation for developing high-performance absorbent pad materials.

### 3.8. Swelling Property

**Figure 7:**
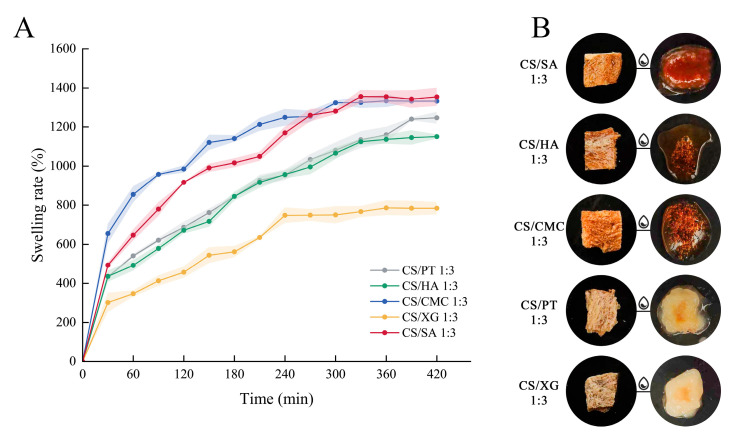
Changes in the swelling property of xerogels with different types of polyanions (**A**) and the appearance of xerogel before and after water absorption (**B**).

The swelling properties of the xerogel directly determine the ability of the material to quickly absorb the meat exudate. Analysis of the swelling kinetic curves (Figure 7) reveals that all xerogel samples exhibited rapid water absorption and swelling during the initial immersion phase, with the degree of swelling increasing sharply within the first 30 min. Subsequently, the swelling rate gradually decreased and eventually reached equilibrium. This dynamic process aligns with the typical swelling mechanism of hydrogels: water initially penetrates the gel network rapidly via capillary action and diffusion, and as the network expands and the polymer chains relax, the swelling rate slows, ultimately reaching a balance between osmotic pressure and network elasticity [53]. Among the five composite xerogels, CS/SA and CS/CMC achieved the highest equilibrium swelling degree, followed by CS/PT and CS/HA, with CS/XG exhibiting the lowest. This difference is mainly due to the charge characteristics, molecular conformation, and network structure formed by different polyanions. SA and CMC molecular chains have high-density carboxyl groups (-COO^−^). At the same time, they can form strong electrostatic crosslinking with the protonated amino groups (-NH^3+^) of CS, constructing a stable and hydrophilic three-dimensional network [54]. Such networks generally exhibit high porosity, offering ample space for rapid water ingress and storage. Although the HA also contains carboxyl groups, its relatively lower charge density results in slightly inferior swelling performance compared to the first two. The limited swelling capacity of CS/PT may be attributed to partial esterification of its carboxyl groups and its branched-chain structure [55], both of which reduce its effective charge density and hydrophilicity. The lowest swelling degree observed in CS/XG is primarily due to its unique rigid helical molecular conformation and extremely high molecular weight. These characteristics significantly increase fluid resistance and form a dense network in solution, severely hindering water penetration and diffusion [56]. It should be noted that excessive swelling may also compromise structural stability. As shown in Figure 7B, after being soaked in water for 8 h, five xerogel samples underwent irreversible destruction of their external shapes and could not maintain their original shapes. In pure water, the extremely low ionic strength can create a large osmotic pressure difference between the interior and exterior of the gel network, potentially leading to over-swelling and irreversible damage to the network structure [57]. Therefore, achieving a balance between swelling capacity and mechanical integrity is essential for practical applications. In brief, CS/SA and CS/CMC, owing to their high charge density and ability to form stable porous networks, exhibited the most favorable swelling performance.

### 3.9. Water Retention Capacity

**Figure 8:**
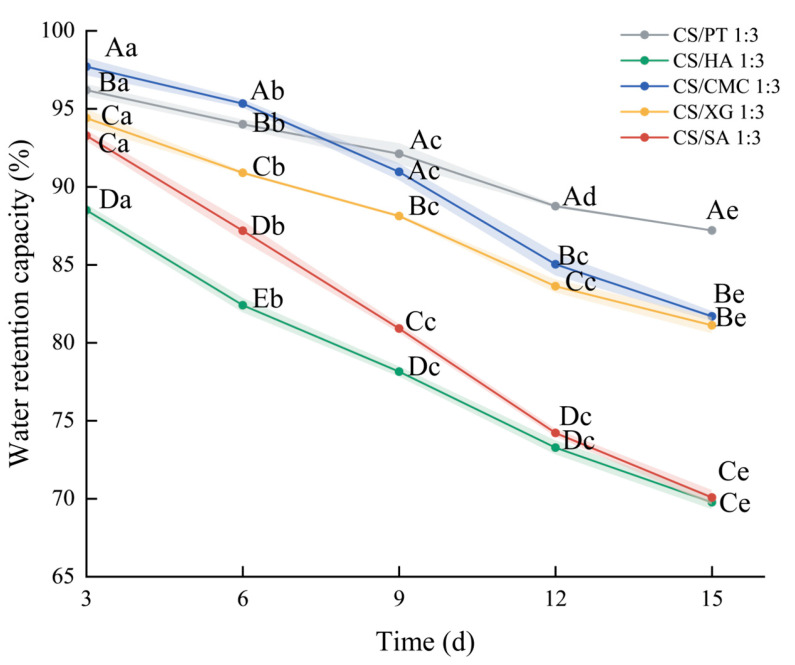
Changes in the water retention capacity (%) of xerogels with different types of polyanions. Different capital letters denote statistically significant differences in the shrinkage ratio among different groups (*p* < 0.05), whereas different lowercase letters indicate statistically significant differences in the water absorption among different groups (*p* < 0.05).

The water retention capacity of dry gel is to evaluate whether the material can effectively lock the absorbed water and prevent the reverse migration of water after absorbing the exudate. As shown in Figure 8, during the initial water-retention phase (0–2 days), all samples exhibited a relatively rapid moisture release rate. This phenomenon is primarily attributed to the migration of internal moisture through the porous network to the surface, followed by evaporation driven by capillary forces and the vapor-pressure difference with the environment [48]. The moisture release rate gradually decreased over time. This phenomenon may be because the bound water is more tightly held by hydrophilic groups and thus harder to desorb.

Among the five xerogels, CS/PT sample exhibited the most excellent water retention performance, with its water retention rate significantly higher than those of the other groups. This can be primarily attributed to the potential formation of microcrystalline regions (Figure 5D) on the surface during drying. These crystalline structures effectively hinder the outward diffusion of internal moisture. CS/CMC and CS/XG samples displayed second-highest water retention rates. CS/CMC relies on the electrostatic repulsion of CMC molecular chains in the hydrated state to maintain network stability. The flexibility of its segments enables stress dissipation through conformational adjustments after water absorption, thereby preventing structural collapse, thus increasing water retention capacity [56,58]. Comparatively, CS/SA and CS/HA samples exhibited a higher degree of network degradation after water absorption, resulting in a network structure with poor stability and easy pore collapse, leading to rapid water loss. Moreover, highly swellable materials, such as CS/SA and CS/HA, despite their strong initial liquid absorption capacity, tend to form a gel structure with insufficient mechanical strength after swelling (Figure 7B). Such a structure is more susceptible to damage under environmental stresses, which compromises long-term water retention [57]. In general, CS/PT exhibited the highest water retention capacity, while CS/CMC achieved a favorable balance between swelling and water retention.

### 3.10. Water Vapor Adsorption Capacity

**Figure 9:**
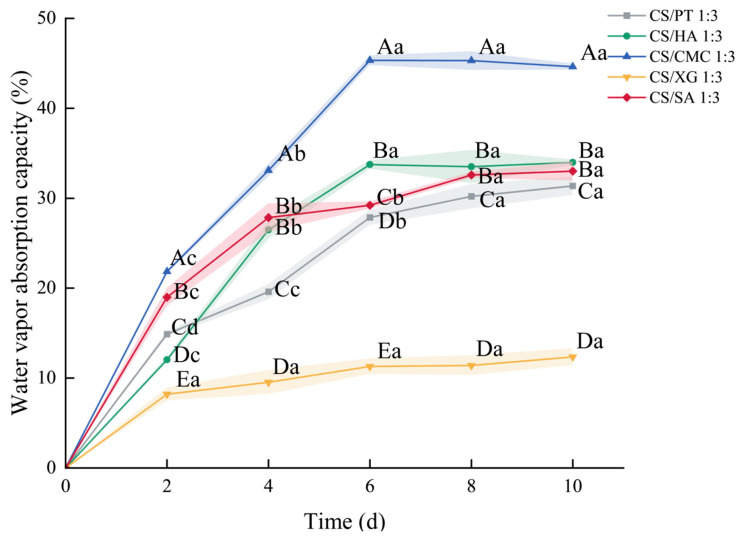
Changes in the water vapor adsorption capacity of xerogels with different types of polyanions. Different capital letters denote statistically significant differences in the shrinkage ratio among different groups (*p* < 0.05), whereas different lowercase letters indicate statistically significant differences in the water absorption among different groups (*p* < 0.05).

The water vapor adsorption capacity of a xerogel reflects its ability to regulate environmental humidity [59]. As shown in Figure 9, the moisture absorption of all samples exhibited a characteristic of initial rapid absorption followed by gradual slowing down. All xerogel groups reached their maximum water vapor adsorption capacity on day 6. The underlying reasons for this trend are as follows: During the initial absorption stage, the abundance of hydroxyl groups on the polyanion molecular chains plays a dominant role, imparting hydrophilicity to the xerogels [47]. Water vapor rapidly enters the pores and binds to surface active sites, where water molecules are adsorbed and liquefied. Over time, as the adsorption capacity of the surface layer approaches saturation, the adsorption rate gradually decreases. Subsequently, internal diffusion, primarily driven by capillary action within the hydrophilic xerogel network, becomes the dominant process. This allows water molecules to continuously permeate the network, facilitating the ongoing adsorption and liquefaction of water vapor as well as the storage of liquid water [32], the water vapor adsorption capacity was highest in CS/CMC, followed by CS/HA, CS/SA, and CS/PT, with CS/XG exhibiting the lowest. The CS/CMC sample exhibited the highest water vapor adsorption capacity, attributable to its high porosity and abundance of hydrophilic groups (Figure 6). Moreover, the toughness of its molecular chains helps maintain the integrity of the outer gel structure upon water absorption, allowing water molecules to continuously penetrate into the gel interior. CS/HA and CS/SA swell more readily upon water vapor absorption, potentially forming more closed-pore structures. This reduces the effective adsorption area and hinders subsequent water ingress into the gel interior. CS/SA exhibited an initial fast followed by a slow absorption rate, while CS/HA maintained a consistently fast absorption rate. This phenomenon may be attributed to the comparatively high porosity of CS/HA, which endows the xerogel with a larger specific surface area, thereby continuously providing binding sites during the adsorption process, thereby continuously providing binding sites during adsorption [60]. For CS/PT, partial methyl esterification of the carboxyl groups on PT reduces the number of effective hydrophilic sites [61]. In addition, its lower porosity is also the reason for its relatively low water vapor adsorption capacity [62]. CS/XG possesses the fewest hydrophilic groups and the lowest porosity. Furthermore, the helical conformation of XG molecules increases the viscosity of the material upon water absorption that impedes water adsorption. Consequently, CS/XG exhibited the poorest water vapor adsorption performance. Generally speaking, CS/CMC sample exhibited superior water vapor adsorption performance compared to the other samples.

### 3.11. Tensile Property

**Figure 10:**
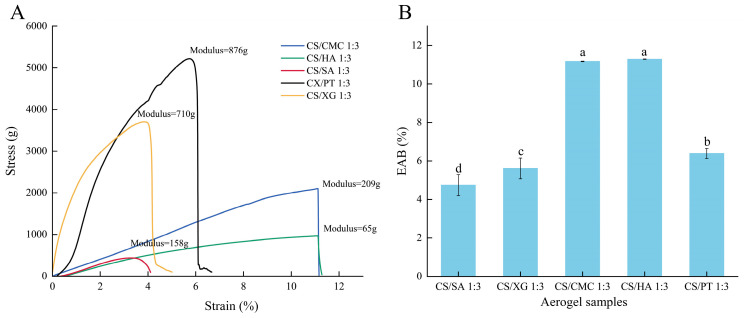
Changes in the tensile property of xerogels with different types of polyanions (**A**) and the EAB of xerogels with different types of polyanions (**B**). Different lowercase letters indicate statistically significant differences in the EAB among different groups (*p* < 0.05).

The tensile property of dry gel is to evaluate the ability of materials to effectively withstand external tensile stress during packaging, stacking and transportation. As shown in Figure 10, the tensile force of all the xerogel samples increased with strain, significant differences were observed in the fracture points and curve shapes, CS/CMC and CS/HA displayed the highest elongation at break, while CS/SA presented the lowest value. CS/XG and CS/PT samples exhibited higher rigidity due to their higher modulus, whereas CS/CMC, CS/SA, and CS/HA showed lower rigidity. CS/SA, CS/XG, and CS/PT samples fractured at strains of approximately 4.1%, 5%, and 6.7%, respectively, indicating limited toughness. Among these, CS/SA fractured early, likely due to its relatively loose gel network and poor continuity of pore walls, which tend to promote stress concentration under load [63]. CS/XG and CS/PT, constrained by the high rigidity of their molecular chains, can withstand higher tensile forces, but their deformation capacity is restricted, displaying typical brittle behavior. In contrast, CS/CMC and CS/HA samples demonstrated superior toughness, as evidenced by higher tensile strengths in their stress–strain curves. This characteristic is primarily attributed to their unique molecular network designs: CMC chains possess excellent flexibility and entanglement capability, allowing them to dissipate energy through chain slippage and rearrangement under stress [64]. HA, with its long-chain hydrophilic structure, forms a dynamically cross-linked network with CS, achieving toughening through the breakage and reformation of reversible bonds during tensile loading [65]. These mechanisms collectively endow the materials with high toughness and damage tolerance [66,67]. In a word, CS/CMC sample, which maintains relatively high tensile strength while offering excellent toughness, is more suitable for packaging scenarios involving repeated stress or deformation.

### 3.12. Formation Mechanism of Chitosan/Polyanion Xerogel

**Figure 11:**
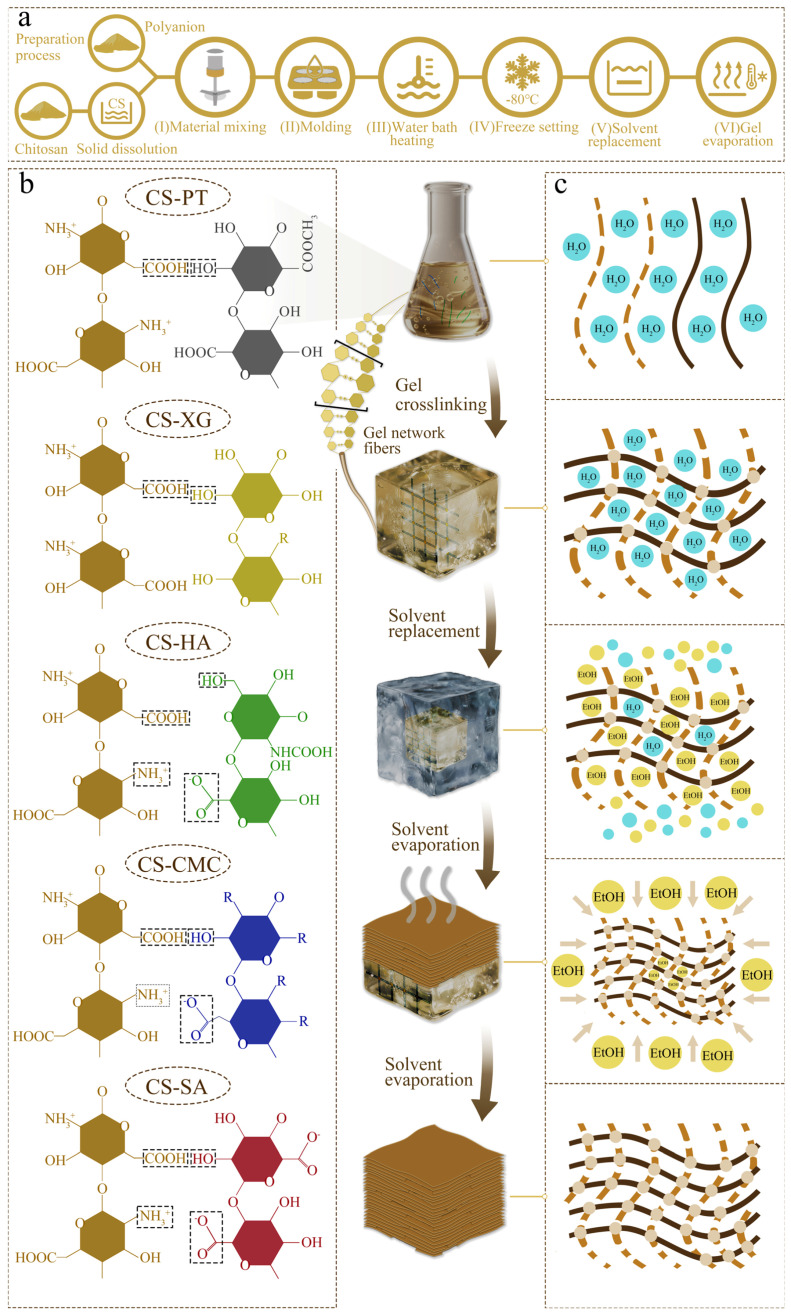
Schematic illustration of the xerogel preparation strategy and key steps. The arrows indicate the processes that the samples undergo. (**a**) Flowchart of the xerogel production process: (I) Raw material mixing; (II) Casting into mold; (III) Heating in a water bath; (IV) Freezing at −80 °C for solidification; (V) Solvent exchange; (VI) Drying. (**b**) Schematic representation of the intermolecular cross-linking between CS and polyanion chains. Different colors represent different polyanions. (**c**) Schematic diagram illustrating the evolution of the gel network density and solvent during the sample preparation process.

The phase transition process of the gel is illustrated in Figure 11. Initially, chitosan is uniformly dispersed in an acidic solution, where its amino groups are protonated to form positively charged moieties (-NH_3_^+^) [68]. Upon addition of the polyanion, under water bath heating and stirring, the negatively charged groups (-COO^−^) on the polyanion rapidly interact with the protonated amino groups via electrostatic attraction and amidation reactions, leading to the gelation and formation of a polyelectrolyte complex. This interaction promotes the transition of chitosan chains from a disordered conformation to an ordered cross-linked network, resulting in gradual gelation and ultimately yielding a hydrogel with a 3D network structure [69]. Subsequently, the hydrogel was frozen at −80 °C for 8 h. During the freezing process, the ice crystals formed in the hydrogel pushed the cellulose nanofibers toward the ice grain boundaries, leading to polymer aggregation and the formation of a network within the frozen gel. Concurrently, the lower freezing temperature and extended freezing duration contributed to the formation of smaller pores. This aggregation process enhanced the mechanical strength of the resulting network structure [70,71]. The frozen hydrogel is then immersed in absolute ethanol for solvent exchange. Ethanol molecules diffuse into the gel network and progressively replace the water molecules. In hydrogel systems, water, as a highly polar solvent, forms a strong hydration layer with hydrophilic groups (e.g., -OH, -COOH, -NH_2_) present in the polymer network. This hydration layer not only facilitates the full extension of polymer chains but also enhances their conformational flexibility through effective solvation, resulting in a porous, loose, and soft three-dimensional network structure [72]. When ethanol is employed to replace water in the hydrogel, its solvation capacity toward hydrophilic groups is markedly reduced. This results in the disruption of the original hydration layer, thereby exposing and intensifying the hydrophobic interactions and hydrogen bonding between the polymer chains. The dehydration increases the rigidity of the molecular chains and restricts the mobility of chain segments, which induces chain contraction and aggregation. Consequently, the gel network undergoes significant densification due to the enhanced intermolecular interactions [73]. Through three rounds of solvent exchange [10], the aqueous phase within the pores is replaced by absolute ethanol, which has a lower surface tension. This process significantly reduces the interaction between the solvent and the gel matrix, thereby minimizing the risk of structural collapse caused by capillary contraction during subsequent drying [10]. Moreover, the high volatility of ethanol facilitates rapid solvent removal, further preserving the micro-morphology of the gel. This process results in a dry gel material with a well-defined three-dimensional structure. Compared to CS/HA, CS/SA, and CS/CMC samples that are cross-linked synergistically through both hydrogen bonds and amide bonds, CS/PT and CS/XG samples primarily rely on weaker hydrogen bonds to form their network structures. The strength provided by hydrogen bonding alone is limited, resulting in weaker tensile resistance and a tendency toward brittle fracture under external stress. In contrast, the introduction of amide bonds enables cooperative interaction with hydrogen bonds, creating a cross-linked network that integrates both rigidity and flexibility, thereby leading to a more ductile gel structure.

The properties of the xerogels are also significantly influenced by the molecular configuration and side-chain groups of the polymers. Molecules with complex branched structures, such as PT and XG, exhibit a “dendritic” topology, facilitating the formation of networks with a “multi-point support” characteristic. This imparts excellent compressive resistance to the material, although the tensile strength remains relatively low. Meanwhile, the steric hindrance caused by their branched chains restricts the swelling of the network upon water absorption. In comparison, molecules with fewer branches, such as SA, HA, and CMC, tend to form more ordered and tightly arranged structures, where the synergistic effect between amide bonds and hydrogen bonds is stronger, leading to superior tensile performance. However, their resistance to compression is comparatively weaker. Additionally, the larger network gaps formed by these molecular chains allow for fuller expansion of the segments during water absorption, endowing them with a higher water absorption capacity.

## 4. Conclusions

This study systematically compared the properties of xerogels prepared from CS and five different polyanions. The results demonstrate that under the optimized CS-to-polyanion mass ratio of 1:3, CMC exhibited distinct advantages. The CS/CMC xerogel formed a stable three-dimensional network, demonstrating the lowest shrinkage and highest porosity while retaining appropriate mechanical strength. Fourier-transform infrared spectroscopy confirmed strong electrostatic interactions and potential amide-bond cross-linking within the system. X-ray diffraction and scanning electron microscopy further revealed its homogeneous, amorphous, and highly porous structure. Compared to the other polyanion systems, CS/CMC xerogel achieved the most favorable overall balance in water absorption capacity, swelling behavior, water vapor adsorption, and mechanical toughness. This combination of superior properties stems from the high carboxyl group density and molecular chain flexibility of CMC, which enables the construction of a network that is both stable and structurally adaptable.

## Data Availability

The original contributions presented in this study are included in the article. Further inquiries can be directed to the corresponding author.
